# Identifying Causal Risk Factors for Violence among Discharged Patients

**DOI:** 10.1371/journal.pone.0142493

**Published:** 2015-11-10

**Authors:** Jeremy W. Coid, Constantinos Kallis, Mike Doyle, Jenny Shaw, Simone Ullrich

**Affiliations:** 1 Violence Prevention Research Unit, Wolfson Institute of Preventive Medicine, Queen Mary University of London. Garrod Building, Turner Street, London E1 2AD, United Kingdom; 2 Institute of Brain, Behaviour and Mental Health, University of Manchester. Oxford Road, Manchester M13 9PL, United Kingdom; University of Geneva, SWITZERLAND

## Abstract

**Background:**

Structured Professional Judgement (SPJ) is routinely administered in mental health and criminal justice settings but cannot identify violence risk above moderate accuracy. There is no current evidence that violence can be prevented using SPJ. This may be explained by routine application of predictive instead of causal statistical models when standardising SPJ instruments.

**Methods:**

We carried out a prospective cohort study of 409 male and female patients discharged from medium secure services in England and Wales to the community. Measures were taken at baseline (pre-discharge), 6 and 12 months post-discharge using the Historical, Clinical and Risk-20 items version 3 (HCR-20^v3^) and Structural Assessment of Protective Factors (SAPROF). Information on violence was obtained via the McArthur community violence instrument and the Police National Computer.

**Results:**

In a lagged model, HCR-20^v3^ and SAPROF items were poor predictors of violence. Eight items of the HCR-20^v3^ and 4 SAPROF items did not predict violent behaviour better than chance. In re-analyses considering temporal proximity of risk/ protective factors (exposure) on violence (outcome), risk was elevated due to violent ideation (OR 6.98, 95% CI 13.85–12.65, P<0.001), instability (OR 5.41, 95% CI 3.44–8.50, P<0.001), and poor coping/ stress (OR 8.35, 95% CI 4.21–16.57, P<0.001). All 3 risk factors were explanatory variables which drove the association with violent outcome. Self-control (OR 0.13, 95% CI 0.08–0.24, P<0.001) conveyed protective effects and explained the association of other protective factors with violence.

**Conclusions:**

Using two standardised SPJ instruments, predictive (lagged) methods could not identify risk and protective factors which must be targeted in interventions for discharged patients with severe mental illness. Predictive methods should be abandoned if the aim is to progress from risk assessment to effective risk management and replaced by methods which identify factors causally associated with violence.

## Introduction

The development of structured risk assessment for violent and antisocial behaviour has been a major advance in clinical practice, both within the mental health and the criminal justice system. More recently, however, there have been fewer developments in the field, with division between adherents to actuarial methods of assessment and Structured Professional Judgement (SPJ) [1–4]. A substantial number of risk assessment instruments have been developed, aimed to help clinicians when making decisions, but with little convincing evidence of superiority of any new instruments over their predecessors [5, 6]. Assessment of predictive accuracy using AUC values has been the standard measure of utility and is almost exclusively used in standardisation and validation of both actuarial and SPJ instruments. The risk factors are typically measured once and then used to statistically predict the occurrence of violence in a subsequent time window with its length substantially varying from study to study. This method assures temporal ordering of risk factor (exposure) and violent behaviour (outcome) but does not take into consideration that some risk factors change and may no longer be in operation when the outcome is measured. Furthermore, predictive accuracy rarely exceeds a moderate level of precision [5] and clinicians must expect three out of ten predictions to be incorrect even when using the best performing instruments. Moreover, a reduction in accuracy (shrinkage) typically occurs when an instrument is subsequently administered in a population on which it was not standardised [7]. Most instruments are checklists where a minority of items have predictive power on which their predictive efficacy ultimately depends [8, 9]. Despite their widespread use, only one randomised controlled trial has been carried out so far which has failed to demonstrate a reduction of violence following administration of an SPJ instrument [10]. There is currently no evidence that violence can be prevented using a standardised risk assessment [10, 11].

Taking into consideration these limitations, it is questionable whether statistical prediction is the most appropriate clinical goal when using a risk assessment instrument if the aim is to intervene to prevent violence. Successful management of risk can only be achieved by targeting risk factors causally related to violence. Surprisingly, few studies have attempted to investigate causal associations between risk factors and violence. Risk factors can be classified as static or dynamic. Whilst no change will occur in the static risk (e.g. gender, ethnicity), it is the nature of a dynamic risk factor to change and fluctuate over time, with varying speed. The risk factor may therefore no longer be present in a subsequent time window when violence is measured. In a predictive (lagged) model, where the risk factor occurs some time before the outcome, significant associations may consequentially be missed. To identify associations which can be considered causal, temporal proximity (co-occurrence) between the risk factor and outcome is of utmost importance [12–14]. This can be difficult to establish and requires specification of a definite time frame when exposure and outcome occur. However, it may not be possible to establish temporal ordering within this time frame and, therefore, associations detected in this specified time window have to be interpreted based on plausibility and available knowledge of cause and effect.

Furthermore, causal risk factors can be poor predictors. For example, persecutory delusions did not demonstrate an association with violence among discharged patients with psychotic illness when the exposure measured in the past ten weeks was used to predict the outcome in the subsequent ten weeks. When considering their co-occurrence in the same ten-week window, there was a substantial and robust association although the effect was mediated by anger due to the delusional beliefs [13–15].

A treatment or risk management intervention which targets a predictor variable, even if it has a high level of accuracy, cannot succeed in preventing violence unless it is also causally related. If the intention is to reduce risk by improving risk management, then causal factors must be accurately identified and subsequently targeted for intervention. To demonstrate the advantages of causal compared to predictive models for the implementation of clinical risk management we carried out a prospective longitudinal study of patients discharged from medium secure services in England and Wales over a twelve-month period. The overall aim of the study was to compare the utility of predictive and causal models when identifying dynamic risk factors that should be targeted in future interventions. To this purpose we (i) examined predictive efficacy of total scores and individual items using two SPJ instruments, one measuring risk, the other protective factors, on violent outcome by using the risk/ protective factors measured as present in the past six months to predict violence in the subsequent six months (lagged model); (ii) we then tested causal models, including the same dynamic variables, in which we considered their co-occurrence within the same six months’ time window (temporal proximity). Since causal pathways to violent behaviour are not always direct, i.e. some associations are explained by a third variable which is associated with both exposure and outcome, we (iii) performed statistical analyses to identify explanatory variables which are the main drivers in the association with violence.

## Methods

The method of data collection has previously been described [16]. In brief, a prospective cohort follow-up study was carried out on all forensic patients discharged from 32 National Health Service (NHS) medium secure units across England and Wales (in 26 NHS secondary care Trusts) between 1^st^ September 2010 and the 31^st^ August 2011. These patients had been detained under compulsory orders following violent and criminal behaviour. Patients who were discharged to the community rather than prison were eligible for the follow-up study. ‘Community’ placements for the purpose of the study included accommodation such as independent tenancies, supported accommodation, hostels, open rehabilitation wards, and open psychiatric units.

A link person was identified at each hospital site and a notification system set up so that researchers would be automatically informed when a patient was discharged. Baseline assessments were then conducted for those discharged by interviewing a member of staff who knew the patient well, and a review of clinical and criminal records. The patients were twice followed-up after release at six and twelve months.

The North West England multi-site research ethics committee approved the study. To ensure a total sample of discharges, permission was sought and granted by the UK National Information Governance Board (NIGB) to conduct the study without patient consent under Section 251 of the NHS Act, 2006.

### Measures

Demographic and diagnostic information (primary diagnosis) was recorded at baseline (pre-release) for each patient. Measures of risk and protective factors were completed based on information from a collateral interview and file review. The following assessments were carried out:

The Historical Clinical Risk Management– 20 items version 3 (HCR-20^v3^) is a violence risk assessment covering a broad range of items. It comprises 10 historical (static) factors, 5 clinical items meant to reflect current, dynamic (changeable) correlates of violence, and 5 risk management items which focus on future factors. The items were scored 0 if not present, 1 if partially present, and 2 if definitely present. Total scores range from 0 to 40.

The Structured Assessment of Protective Factors (SAPROF) is a guideline developed to measure protective factors that mitigate future risk of violence. It includes 17 items covering internal, motivational and external factors. Each item is rated on a 3-point scale (0, 1 or 2) as the HCR-20^v3^. Total scores range between 0 and 34.

Violence was measured using the McArthur community violence instrument (MCVI) which comprises 18 questions focusing on violent incidents not necessarily leading to a caution or conviction. THE MCVI differentiates experiences of violent victimisation and violence perpetration. Violence perpetration was defined by combining ‘violence’ and ‘other aggressive acts’, which could include sexual acts, assaultive acts involving use of a weapon, or threats made with a weapon in the hand, as well as acts of battery, regardless of whether they resulted in injury. Verbal threats alone were not included. As with the HCR-20^v3^ and SAPROF, details of any violent incidents mentioned in the clinical case files were noted and cross-matched with the information gathered at interview with the link person. In addition, details of criminal convictions in the twelve months post-discharge were obtained from the Police National Computer (PNC). An individual was deemed to have offended if they had committed a recordable offence within the follow-up period and had the offence proven later, either by accepting a caution, warning or reprimand, or by being found guilty in a court of law. MCVI ratings of violent perpetration as described above and PNC violent cautions/ convictions were then combined to a measure of “any” violence.

Ratings of the HCR-20^v3^ and SAPROF were carried out at baseline, prior to or shortly after discharge, and at 6 and 12-months post discharge (apart from the historical risk factors of the HCR-20^v3^ which are considered stable and were only assessed at baseline). Measures of violence were taken at 6 and 12-months post-discharge.

Inter-rater reliability was calculated between four raters on 20 cases who had been trained and supervised in the use of all instruments. Raters obtained an IRR of 0.92 on the HCR-20^v3^ and 0.98 on SAPROF total scores.

### Statistical analysis

For the predictive approach (lagged model), risk/ protective factors which occurred in the past 6 months were modelled as statistical predictors for violent outcome occurring in the subsequent 6 months. In the temporal proximity or causal models, both predictors and outcome occurred within the same 6 month time window.

In order to take advantage of the longitudinal design, multilevel modelling was performed. These models account for the dependence of data collected longitudinally by modelling the correlation of repeated measures within the same individual as random effects. Unlike other approaches, such as ANOVA, mixture models do not require that data are complete for individuals at each time point or imputation of data which may result in bias [17].

We firstly assessed associations of individual HCR-20^v3^ and SAPROF items using the Area under the ROC Curve (AUC), a statistic that provides a measure of discrimination for each item between violent and non-violent cases. As stated above, analyses were performed using a predictive/ lagged and a temporal proximity/ causal modelling approach. We used the Stata command somersd to estimate AUC statistics that allows for clustering to be taken into consideration. However, somersd allows only for one level of clustering and we therefore estimated AUC statistics allowing for repeated measures to be nested within subjects (2-level structure).

We then carried out predictive/ lagged and temporal proximity/ causal logistic regression analyses to obtain estimates of the Odds Ratio (OR) for the effects of each risk/ protective factor on violence. These were 3-level mixed effect models with repeated measures (level 1) nested within subjects (level 2) nested within the NHS Trusts responsible for the secure services from where the patients had been discharged (level 3).

Since information on temporal ordering was not available for the risk/ protective factors and it was not possible to plausibly infer the order, we could not carry out mediation analyses. The final set of analyses therefore aimed to identify variables which explained the association of certain risk/ protective factors with violent outcome. In order to qualify as explanatory variable a risk/ protective factor was required to be associated with both exposure (other risk/ protective factors) and violent outcome. Only if both associations were significant at an alpha level of <0.05 were variables selected and then entered in an adjusted model. We examined the percentage reduction in the baseline odds of each risk/ protective factor after adding each of the potentially explanatory variables into the following equation:

$$100\times \frac{\left(\beta_{unadjusted}-\beta_{adjusted}\right)}{\beta_{unadjusted}}$$

In a final model, all explanatory variables were entered simultaneously. Comparisons between baseline-adjusted and fully adjusted coefficients were used to estimate the extent to which the association between a risk/ protective factor and violent outcome was accounted for by the explanatory variable. Percentages above 100 were possible if the OR in the fully adjusted model changed to a value below or above 1. As above, we again performed 3-level mixed effect models with repeated measures (level 1) nested within subjects (level 2) nested within discharging NHS Trusts (level3).

All statistical analyses were carried out in Stata 14. The statistical programming code can be found in S1 Appendix. A significance level of 0.05 was adopted throughout.

## Results

There were 788 patients discharged during the study period, of whom 409 (52%) were discharged to the community. At 6-months post discharge, collateral interview and case note reviews were completed for 387 (95%), and at 12-months for 344 (89%) patients. At 6-months post discharge, 54 (14.0%) patients had committed a violent act, and at 12-months 43 (12.5%) had been violent. It was possible that a patient acted violently in both 6 month time windows.

The mean age of the study participants was 37.8 years (SD = 9.7); 344 (88.9%) men and 43 (11.1%) women; 232 (60.1%) were white, 98 (25.4%) black, 24 (6.2%) South Asian, 24 (6.2%) mixed heritage, and 8 (2.1%) Chinese or of other ethnic origin. Primary diagnoses included 313 (80.9%) schizophrenia/ schizoaffective disorder, 28 (7.2%) bipolar disorder, 21 (5.4%) personality disorder, 3 (3.8%) anxiety disorder, 5 (1.3%) depression, 1 (0.3%) substance use, and 16 (4.1%) other diagnoses.

### Predictive accuracy of static risk factors

As shown in Table 1, only 4 of the 10 individual static risk factors of the historic domain of the HCR-20^v3^ achieved a statistically significant but poor level of accuracy including: other antisocial behaviour, traumatic experiences, violent attitudes and treatment or supervision response. The AUC value for the total score was also significant but predicted violence just above chance. This was reflected by the results of the logistic regression model which identified the same variables as significant predictors of violent behaviour. After adjustment of age, gender, ethnicity and primary diagnosis at baseline, only traumatic experiences and the historical total score remained significant.

**Table 1:** Predictive accuracy of HCR-20^v3^ historical items (measured at baseline).

	AUC ^1)^	95% CI	P	AOR ^2)^	95% CI	P	AOR ^3)^	95% CI	P
Violence	0.49	0.44–0.54	0.736	0.97	0.50–1.87	0.920	0.83	0.43–1.58	0.570
Other antisocial behaviour	0.57	0.52–0.63	0.011	1.55	1.08–2.21	0.017	1.42	0.99–2.04	0.055
Relationships	0.51	0.46–0.57	0.688	1.15	0.74–1.78	0.545	1.22	0.78–1.91	0.376
Employment	0.55	0.49–0.60	0.096	1.43	0.93–2.20	0.103	1.34	0.87–2.06	0.190
Substance use	0.53	0.48–0.58	0.201	1.35	0.90–2.01	0.148	1.19	0.78–1.82	0.430
Major mental disorder	0.49	0.45–0.52	0.484	0.88	0.38–2.04	0.769	1.12	0.47–2.69	0.800
Personality disorder	0.54	0.48–0.60	0.188	1.27	0.90–1.80	0.181	1.24	0.85–1.79	0.262
Traumatic experiences	0.59	0.53–0.65	0.002	1.76	1.19–2.60	0.004	1.58	1.08–2.32	0.020
Violent attitudes	0.59	0.53–0.64	0.003	1.58	1.12–2.24	0.009	1.39	0.99–1.96	0.060
Treatment or supervision response	0.56	0.50–0.62	0.036	1.56	1.05–2.32	0.027	1.38	0.93–2.04	0.105
Total score	0.60	0.54–0.66	0.001	1.15	1.05–1.25	0.002	1.12	1.02–1.22	0.014

^1)^ 2-level mixed effect model (relatedness of measures/ subjects).

^2)^ 3-level mixed effect model (relatedness of measures/ subjects/ Trust).

^3)^ 3-level mixed effect model (relatedness of measures/ subjects/ Trust) adjusted for age, gender, ethnicity, primary diagnosis at baseline. *AUC* area under the ROC curve. *AOR* adjusted odds ratio. *95% CI*: 95 percent confidence interval. *P* level of statistical significance.

### Dynamic risk and protective factors for violence: predictive vs. causal models

**Fig 1:**
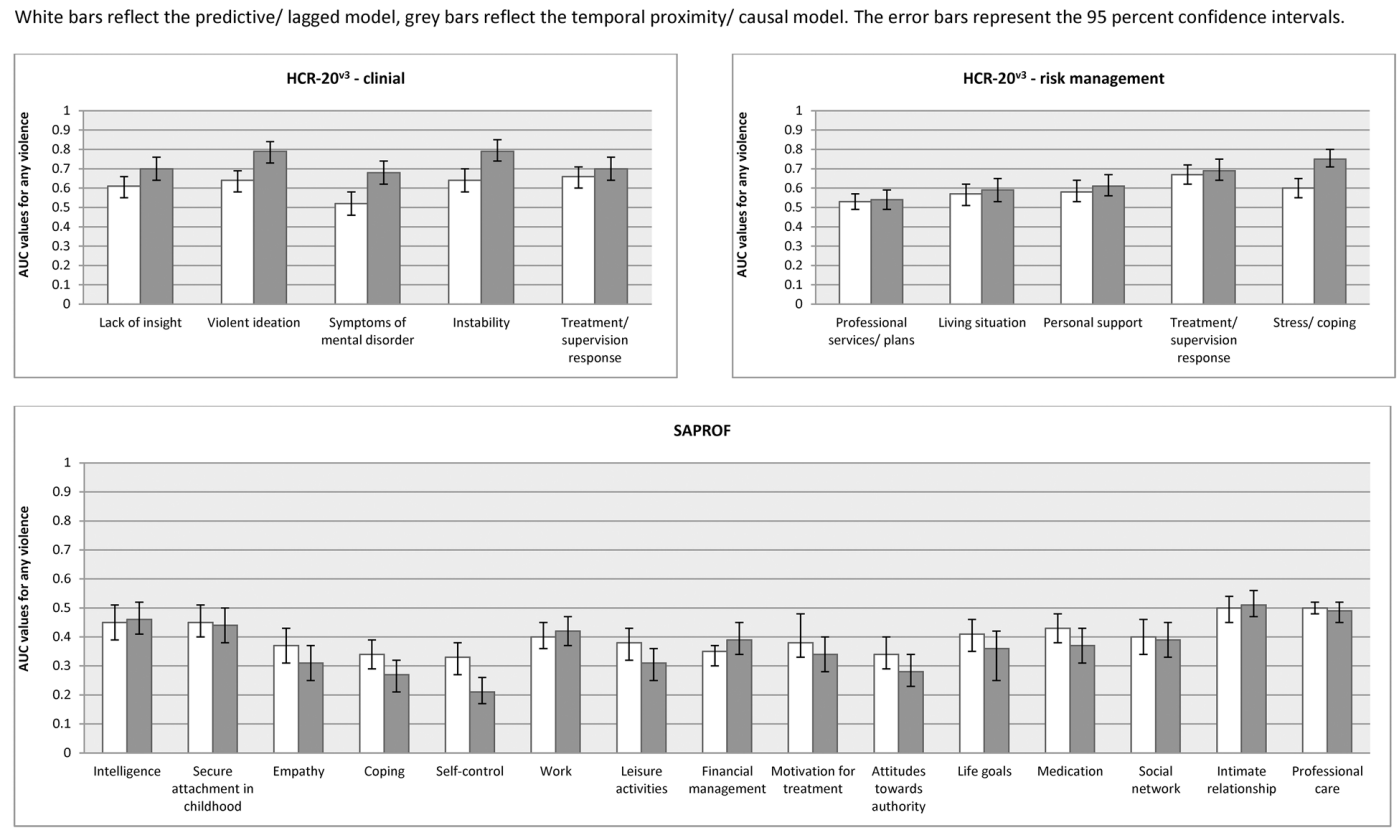
Predictive vs. causal models–accuracy. White bars reflect the predictive / lagged model, grey bars reflect the temporal proximity / causal model. The error bars represent 95 per cent confidence intervals.

The AUC values of the lagged/ predictive and temporal proximity/ causal analyses for the dynamic items of the HCR-20^v3^ and SAPROF are reported in Table 2 and Fig 1. In the predictive model, all dynamic items of the HCR-20^v3^ except symptoms of mental disorder and professional services and plans achieved a significant AUC value although of poor magnitude. In the causal model, only professional services and plans did not reach the threshold of statistical significance. When comparing the 95% confidence intervals of the two models, the AUCs of most of the clinical risk factors were significantly higher (the 95% CIs did not overlap) and of moderate accuracy in the causal model. These included: violent ideation or intent, symptoms of major mental disorder, instability, and the total clinical risk score.

**Table 2:** Predictive accuracy of lagged vs. temporal proximity models—AUC values.

	Lagged/ predictive			Temporal proximity/ causal		
	AUC	95% CI	P	AUC	95% CI	P
**HCR-20** ^**v3**^ **—clinical**						
Lack of insight	0.61	0.55–0.66	<0.001	0.70	0.64–0.76	<0.001
Violent ideation or intent	0.64	0.58–0.69	<0.001	0.79	0.73–0.84	<0.001
Symptoms of major mental disorder	0.52	0.46–0.58	0.475	0.68	0.62–0.74	<0.001
Instability	0.64	0.58–0.70	<0.001	0.79	0.74–0.85	<0.001
Treatment or supervision response	0.66	0.60–0.71	<0.001	0.70	0.64–0.76	<0.001
Total score	0.67	0.61–0.73	<0.001	0.82	0.77–0.87	<0.001
**HCR-20** ^**v3**^ **–risk management**						
Professional services and plans	0.53	0.49–0.57	0.136	0.54	0.49–0.59	0.098
Living situation	0.57	0.51–0.62	0.014	0.59	0.53–0.65	0.003
Personal support	0.58	0.53–0.64	0.002	0.61	0.56–0.67	<0.001
Treatment or supervision response	0.67	0.62–0.72	<0.001	0.69	0.64–0.75	<0.001
Stress or coping	0.60	0.55–0.65	<0.001	0.75	0.71–0.80	<0.001
Total score	0.67	0.61–0.72	<0.001	0.75	0.70–0.81	<0.001
**SAPROF**						
Intelligence	0.45	0.39–0.51	0.078	0.46	0.41–0.52	0.206
Secure attachment in childhood	0.45	0.40–0.51	0.124	0.44	0.38–0.50	0.065
Empathy	0.37	0.31–0.43	<0.001	0.31	0.25–0.37	<0.001
Coping	0.34	0.29–0.39	<0.001	0.27	0.21–0.32	<0.001
Self-control	0.33	0.27–0.38	<0.001	0.21	0.17–0.26	<0.001
Work	0.40	0.36–0.45	<0.001	0.42	0.37–0.47	0.001
Leisure activities	0.38	0.32–0.43	<0.001	0.31	0.25–0.36	<0.001
Financial management	0.35	0.30–0.40	<0.001	0.39	0.34–0.45	<0.001
Motivation for treatment	0.38	0.33–0.43	<0.001	0.34	0.28–0.40	<0.001
Attitudes towards authority	0.34	0.29–0.40	<0.001	0.28	0.23–0.34	<0.001
Life goals	0.41	0.35–0.46	0.001	0.36	0.30–0.42	<0.001
Medication	0.43	0.38–0.48	0.007	0.37	0.31–0.43	<0.001
Social network	0.40	0.34–0.46	0.001	0.39	0.33–0.45	<0.001
Intimate relationship	0.50	0.45–0.54	0.882	0.51	0.47–0.56	0.612
Professional care	0.50	0.48–0.52	0.761	0.49	0.45–0.52	0.465
Living circumstances	0.43	0.38–0.49	0.014	0.50	0.45–0.56	0.902
External control	0.40	0.35–0.45	<0.001	0.49	0.44–0.55	0.834
Total score	0.26	0.21–0.31	<0.001	0.24	0.19–0.29	<0.001

**Note.** 2-level mixed effect model (relatedness of measures/ subjects). *AUC* area under the ROC curve. *95% CI* 95 percent confidence interval. *P* level of statistical significance.

Apart from professional services and plans, all items of the HCR-20^v3^ risk management scale achieved a significant AUC value in the predictive model (Table 2 and Fig 1). The causal model corresponded to these findings. The AUC value of the total score, though, was significantly higher in the causal compared to the predictive model.

With regard to the protective factors of the SAPROF, predictive and causal analyses yielded similar results regarding the magnitude of the AUC values (Table 2 and Fig 1). Intelligence, secure attachment in childhood, intimate relationship and professional care failed to achieve a significant AUC value in the predictive model compared to intelligence, secure attachment in childhood, intimate relationship, professional care, living circumstances and external control where the AUCs did not reach statistical significance in the causal model. The AUC value of self-control was significantly higher in the causal model.

**Fig 2:**
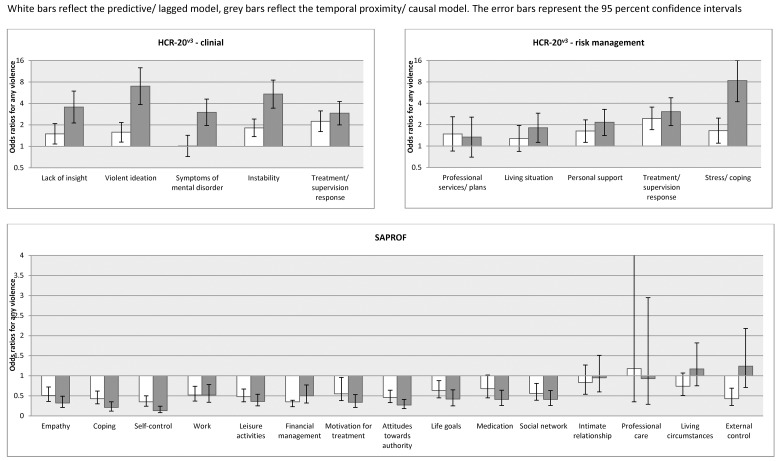
Predictive vs. causal models–strength of association. White bars reflect the predictive / lagged model, grey bars reflect the temporal proximity / causal model. The error bars represent 95 per cent confidence intervals.

In the logistic regression model, all HCR-20^v3^ clinical items were significantly associated with violence in the predictive model with the exception of symptoms of major mental disorder. This risk factor, however, was significant using the causal modelling approach. Apart from treatment or supervision response, all ORs were significantly greater in magnitude in the causal model (Table 3 and Fig 2).

**Table 3:** Strength of association in lagged vs. temporal proximity models–odds ratios.

	Lagged/ predictive			Temporal proximity/ causal		
	AOR	95% CI	P	AOR	95% CI	P
**HCR-20** ^**v3**^ **—clinical**						
Lack of insight	1.50	1.08–2.09	0.015	3.56	2.13–5.96	<0.001
Violent ideation or intent	1.58	1.15–2.16	0.004	6.98	3.85–12.65	<0.001
Symptoms of major mental disorder	1.01	0.72–1.43	0.942	3.00	1.96–4.61	<0.001
Instability	1.82	1.37–2.42	<0.001	5.41	3.44–8.50	<0.001
Treatment or supervision response	2.25	1.61–3.14	<0.001	2.92	2.00–4.27	<0.001
Total score	1.20	1.10–1.30	<0.001	1.67	1.45–1.91	<0.001
**HCR-20** ^**v3**^ **–risk management**						
Professional services and plans	1.48	0.85–2.59	0.166	1.34	0.70–2.56	0.380
Living situation	1.28	0.84–1.95	0.246	1.81	1.13–2.91	0.013
Personal support	1.63	1.13–2.34	0.009	2.16	1.41–3.29	<0.001
Treatment or supervision response	2.45	1.70–3.54	<0.001	3.05	1.94–4.79	<0.001
Stress or coping	1.65	1.10–2.48	0.015	8.35	4.21–16.57	<0.001
Total score	1.30	1.15–1.47	<0.001	1.59	1.37–1.85	<0.001
**SAPROF**						
Empathy	0.51	0.36–0.72	<0.001	0.32	0.21–0.49	<0.001
Coping	0.43	0.30–0.62	<0.001	0.21	0.12–0.35	<0.001
Self-control	0.35	0.24–0.50	<0.001	0.13	0.08–0.24	<0.001
Work	0.52	0.37–0.74	<0.001	0.52	0.34–0.78	0.002
Leisure activities	0.48	0.35–0.67	<0.001	0.36	0.25–0.54	<0.001
Financial management	0.35	0.23–0.52	<0.001	0.50	0.32–0.77	0.002
Motivation for treatment	0.55	0.38–0.79	0.001	0.34	0.21–0.53	<0.001
Attitudes towards authority	0.46	0.33–0.64	<0.001	0.27	0.18–0.41	<0.001
Life goals	0.63	0.45–0.88	0.007	0.42	0.27–0.65	<0.001
Medication	0.68	0.45–1.02	0.062	0.41	0.26–0.64	<0.001
Social network	0.56	0.39–0.81	0.002	0.41	0.26–0.63	<0.001
Intimate relationship	0.83	0.54–1.27	0.384	0.95	0.60–1.51	0.821
Professional care	1.18	0.35–4.02	0.792	0.93	0.29–2.95	0.905
Living circumstances	0.74	0.51–1.07	0.105	1.17	0.75–1.82	0.483
External control	0.43	0.26–0.69	<0.001	1.24	0.71–2.18	0.455

**Note.** 3-level mixed effect model (relatedness of measures/ subjects/ Trust) adjusted for age, gender, ethnicity, primary diagnosis at baseline. *AOR* adjusted odds ratio. *95% CI* 95 percent confidence interval. *P* level of statistical significance.

Personal support, treatment or supervision response, stress or coping, and the total score in the risk management domain were significantly associated with violent outcome in the predictive model (Table 3 and Fig 2). These risk factors were also significant in the causal model in addition with living situation. Stress or coping demonstrated a significantly stronger association with violence in the causal model.

As shown in Table 3 and Fig 2, the majority of protective factors were inversely associated with violence in the predictive model apart from medication, intimate relationships, professional care, and living circumstances. Variables that were not significantly associated with violence in the causal model included intimate relationships, professional care, living circumstances, and external control. The inverse association with self-control was significantly stronger in the causal compared to the predictive model.

### Identification of explanatory variables

As shown in Table 4, the items of the HCR-20^v3^ (historical and dynamic) and of the SAPROF were only moderately correlated. In order to identify risk and protective dynamic factors independently associated with violent outcome, they were entered simultaneously in the logistic regression model. Table 5 shows that in the baseline model, among the 10 dynamic risk factors of the HCR-20^v3^, only 3 (violent ideation or intent, instability, and stress or coping) demonstrated independent effects on violent behaviour. In model 1, Table 5, the baseline models which resulted in a significant association with violence (Table 3) were next adjusted for the first explanatory variable, violent ideation or intent. Although there was attenuation in the OR of symptoms of major mental disorder, the association was still significant. Inclusion of instability led to substantial attenuation of all ORs in model 2, whereas inclusion of stress or coping in model 3 did not demonstrate explanatory effects in the association between lack of insight, symptoms of major mental disorder, and treatment or supervision response with violent behaviour. Inclusion of all 3 explanatory variables in model 4 resulted in statistically non-significant effects of all variables with a change in ORs of approximately 100%. This indicates that violent/ ideation or intent, instability, and stress or coping fully accounted for the association between lack of insight, symptoms of major mental disorder, treatment or supervision response (clinical), living situation, personal support, and treatment or supervision response (risk management) with violent behaviour.

**Table 4:** Inter-item correlations.

	M	SD	Md	No. significant ^1)^
**HCR-20** ^**v3**^ **historical**				
Baseline	0.14	0.11	0.14	7 # 45
**HCR-20** ^**v3**^ **clinical/ risk management**				
Baseline	0.29	0.13	0.26	26 # 45
6 months follow-up	0.33	0.14	0.30	35 # 45
12 months follow-up	0.35	0.16	0.32	30 # 45
**SAPROF**				
Baseline	0.18	0.16	0.16	32 # 136
6 months follow-up	0.25	0.17	0.25	52 # 105
12 months follow-up	0.23	0.17	0.22	45 # 105

**Note.** Numbers in cells are descriptive statistics of Spearman correlation coefficients between each pair of items.

^1)^ Statistical significance was based on a Bonferroni corrected alpha (0.05 / number of correlations). *M* mean, *SD* standard deviation, *Md* median.

**Table 5:** Explanatory variables—HCR-20^v3^ clinical and risk management variables.

	Temporal proximity / causal			
	AOR	95% CI	P	% change
**Model 0: variables are entered simultaneously**				
Lack of insight	1.17	0.66–2.08	0.589	N/A
Violent ideation or intent	3.36	1.89–5.96	<0.001	N/A
Symptoms of major mental disorder	1.05	0.68–1.63	0.827	N/A
Instability	2.29	1.36–3.85	0.002	N/A
Treatment or supervision response	1.14	0.61–2.15	0.684	N/A
Professional services and plans	0.64	0.30–1.35	0.240	N/A
Living situation	1.06	0.61–1.86	0.828	N/A
Personal support	1.00	0.59–1.68	0.898	N/A
Treatment or supervision response	0.61	0.30–1.26	0.185	N/A
Stress or coping	3.11	1.53–6.33	0.002	N/A
**Model 1: adjusted for violent ideation / intent**				
C: Lack of insight	1.59	0.97–2.59	0.067	63.48
C: Symptoms of major mental disorder	1.68	1.12–2.52	0.012	52.78
C: Treatment or supervision response	1.52	1.00–2.33	0.052	60.93
R: Living situation	1.07	0.64–1.77	0.802	88.60
R: Personal support	1.15	0.70–1.87	0.587	81.85
R: Treatment or supervision response	1.44	0.89–2.32	0.139	67.30
**Model 2: adjusted for instability**				
C: Lack of insight	1.55	0.98–2.44	0.061	65.49
C: Symptoms of major mental disorder	1.38	0.92–2.09	0.122	70.68
C: Treatment or supervision response	1.31	0.86–2.00	0.204	74.80
R: Living situation	1.11	0.69–1.80	0.656	82.41
R: Personal support	1.40	0.90–2.16	0.133	56.31
R: Treatment or supervision response	1.22	0.76–1.94	0.407	82.17
**Model 3: adjusted for stress or coping**				
C: Lack of insight	2.13	1.31–3.46	0.002	40.45
C: Symptoms of major mental disorder	1.88	1.23–2.86	0.003	42.54
C: Treatment or supervision response	1.78	1.19–2.66	0.005	46.19
R: Living situation	1.25	0.77–2.02	0.363	62.39
R: Personal support	1.51	0.96–2.36	0.072	46.49
R: Treatment or supervision response	1.50	0.94–2.41	0.092	63.64
**Model 4: adjusted for all explanatory variables**				
C: Lack of insight	0.99	0.61–1.60	0.961	100.79
C: Symptoms of major mental disorder	1.07	0.71–1.62	0.754	93.84
C: Treatment or supervision response	0.89	0.54–1.45	0.631	110.87
R: Living situation	0.89	0.54–1.47	0.650	119.64
R: Personal support	0.96	0.58–1.58	0.870	105.30
R: Treatment or supervision response	0.71	0.42–1.20	0.200	130.71

**Note.** 3-level mixed effect model (relatedness of measures/ subjects/ Trust) adjusted for age, gender, ethnicity, primary diagnosis at baseline. *AOR* adjusted odds ratio. *95% CI* 95 percent confidence interval. *% change* percentage of change from baseline coefficient to coefficient adjusted for explanatory variable. *P* level of statistical significance.

The same procedure was finally carried out with the items of the SAPROF. As can be seen in Table 6, only self-control demonstrated an independent protective effect on violent outcome (model 0). After adjusting the significant protective factors derived from Table 3, most of these were no longer significant, indicating that self-control was a highly important explanatory factor. Only the effects of empathy, leisure activities, and attitudes towards authority were not explained by self-control. These factors conveyed direct protective effects on violent behaviour.

**Table 6:** Explanatory variables–SAPROF.

	Temporal proximity/ causal			
	AOR	95% CI	P	
**Model 0: all variables are entered simultaneously**				
Empathy	0.75	0.47–1.18	0.213	
Coping	0.89	0.49–1.61	0.693	
Self-control	0.21	0.12–0.39	<0.001	
Work	0.93	0.61–1.41	0.733	
Leisure activities	0.68	0.44–1.04	0.072	
Financial management	1.10	0.70–1.74	0.668	
Motivation for treatment	1.17	0.65–2.10	0.601	
Attitudes towards authority	0.71	0.42–1.19	0.190	
Life goals	1.11	0.70–1.75	0.664	
Medication	1.11	0.68–1.83	0.672	
Social network	0.80	0.50–1.26	0.328	
Intimate relationship	1.46	0.96–2.23	0.078	
Professional care	0.95	0.31–2.90	0.925	
Living circumstances	1.51	0.95–2.39	0.079	
External control	1.14	0.68–1.90	0.620	
**Model 1: adjusted for self-control**				**% change**
Empathy	0.62	0.41–0.92	0.018	58.05
Coping	0.65	0.37–1.14	0.131	72.40
Work	0.80	0.53–1.19	0.271	65.88
Leisure activities	0.66	0.45–0.98	0.037	59.33
Financial management	1.08	0.68–1.70	0.749	111.10
Motivation for treatment	0.85	0.54–1.36	0.506	84.94
Attitudes towards authority	0.60	0.39–0.92	0.020	60.99
Life goals	0.87	0.57–1.31	0.494	83.95
Medication	0.90	0.58–1.39	0.620	88.18
Social network	0.70	0.46–1.08	0.111	60.00

**Note.** 3-level mixed effect model (relatedness of measures/ subjects/ Trust) adjusted for age, gender, ethnicity, primary diagnosis at baseline. *AOR* adjusted odds ratio. *95% CI* 95 percent confidence interval. *% change* percentage of change from baseline coefficient to coefficient adjusted for explanatory variable. *P* level of statistical significance.

## Discussion

The standard approach to validation of SPJ instruments employs a predictive method, with corresponding statistical tests to demonstrate predictive accuracy. Accuracy is typically measured using the AUC statistic and, less commonly, methods used to evaluate medical screens for diseases [18–21]. Using this approach, we were unable to validate either the HCR-20^v3^ or SAPROF (using their individual items and total scores) in a representative sample of UK patients discharged from medium security who had committed serious offences or had been unmanageable in other hospital services due to their violence. Items in these instruments were poor at discriminating which patients would become violent over the next 12 months and showed low levels of statistical association with outcome. The predictive method did not therefore indicate which risk factors should be targeted for future risk management or treatment interventions if the aim is to prevent violence. We also observed that the HCR-20^v3^ performed more poorly than in previous studies using the HCR-20^v2^ [5] despite its recent revision aimed to improve its clinical utility. Similarly, few items in the SAPROF showed any discriminative value in identifying patients who would not become violent, with low levels of association between each item and no reported violence over a 12 month period. Nevertheless, consideration of temporal proximity of risk/ protective factors and violence indicative of a causal relationship demonstrated better clinical utility in terms of accuracy and strength of association with violence. We found that the predictive approach substantially underestimated the effects of violent ideation or intent, symptoms of major mental disorder and instability as risk, and self-control as protective, factors for violent behaviour. Their AUC values in the causal model were significantly higher than in the predictive model and achieved satisfactory accuracy. This was confirmed in logistic regression models testing the strength of association where the same variables conveyed significantly higher risk/ protective effects on violent outcome and should be therefore considered as important factors for intervention.

It is of interest that the majority of the variables that were strongly discriminative and associated with violence stemmed from the clinical domain of the HCR-20^v3^. Recent research has demonstrated that most changes in HCR-20^v2^ scores are clinically irrelevant and that only the score of the clinical domain reduced over repeated risk assessments over time [22]. Our findings suggest that this effect continues with the 3^rd^ version of the HCR-20 since the majority of risk management items showed similar discriminative effects and strength of association in the predictive and the causal models. It appears that there are only small changes in most of these items over a follow-up period of 12 months. We also observed the same pattern with the items of the SAPROF and where only self-control demonstrated significantly better discriminative effects and was a stronger protective factor in the causal model. This suggests that the majority of items in the SAPROF are relatively stable over time.

### Explanatory factors for violence

We confirmed that the majority of risk and protective factors were in operation during follow-up, significantly associated with violence, and could therefore have exerted causal effects on violence. However, because only violent ideation or intent, instability, and stress/poor coping were independent risk factors, and only self-control was independently protective, it was unlikely that the other risk and protective factors we identified had exerted their effects directly on violence.

Analysis confirmed that poor insight, symptoms of major mental disorder, poor treatment response, low level of personal support, and unsatisfactory living situation all increased risk of violence, but only when accompanied by violent ideation, behavioural instability, and stress. These latter factors appeared to be explanatory factors in our statistical analysis. They should therefore be key targets for intervention in risk management. Nevertheless, all factors were closely linked and violent ideation, behavioural instability, and stress may have been the outcome of combinations of other factors involving deteriorating mental state, lack of insight, poor response to treatment, and lack of support whilst in an unsatisfactory living situation, all of which require preventive intervention.

Similarly, empathy, coping, work, leisure activities, good financial management, motivation for treatment, positive attitudes towards authority, life goals, taking medication, and positive social networks all conveyed protective effects and reduced the risk of violence, but only when accompanied by good self-control. The explanatory effect of just one variable on these other factors was unexpected and requires further consideration. Good self-control would appear a broad and, arguably, an over-inclusive factor to include in a checklist of protective items, potentially overlapping with certain factors we found were no longer associated with violence after adjusting for each other. Most importantly, the converse of good self-control is poor self-control, as indicated by a history of previous violence in this study.

### Limitations

Our study relied on data collected from extensive case files. Baseline and follow-up interviews were conducted with collaterals who were professional staff currently working with the patients with good knowledge of their past history, recent mental state and functioning. However, absence of a direct patient interview is a limitation of this approach. Nevertheless, this method was deliberately chosen to prevent attrition and where a substantial subgroup of patients reported they would not have co-operated in face-to-face interviews or follow-up, a proportion of whom were subsequently violent. This method, which did not require patient consent under UK legislation, minimised loss to follow-up and resulting biases.

We identified explanatory variables by entering them simultaneously in the statistical model and, therefore, exceeded the recommended ratio of 1 covariate per 10 events. As a consequence, we cannot rule out the possibility of over-fitting in these models. Larger samples with multiple measures of violent outcome are necessary to confirm our findings.

When investigating relationships between dynamic risk/ protective factors and violence, it is essential to account for the fact that both exposure and outcome are dynamic in nature and fluctuate over time. It has been emphasized that temporal proximity is of utmost importance to uncover such associations [12–14]. However, it can be argued that temporal proximity does not necessarily imply temporal ordering and, therefore, the possibility of reversed causality has to be taken into consideration. Criteria of causality were published some decades ago [23] and have been the guidelines in epidemiology. These include temporal ordering of exposure and outcome. However, another criterion is plausibility and (from a clinical perspective) it is more plausible that the risk and protective factors identified in our study lead to an increase/ reduction in violence rather than the reverse pathway.

### Implications

To our knowledge, this is the first study to compare predictive and causal models of association between risk and protective factors, measured using SPJ instruments, in a prospective follow-up study. We found that prediction of future violence has very limited usefulness in a clinical risk assessment. Assessment of predictive accuracy cannot facilitate the identification of the most important risk factors that must be targeted to prevent violence. It also questions whether developing new instruments which are validated using predictive methods [8, 9] should continue in future.

Moving to causal models, which in turn lead to improved clinical interventions and ultimately violence prevention, is necessary for improved service provision and clinical performance. Patients in the offender population we studied have shown increasing length of stay. The throughput of patients admitted to secure forensic services in the UK and other European countries has progressively declined over the past two decades. This follows increasing political and other external pressures against discharging offender patients within increasingly risk-adverse European societies. Emphasis on risk without corresponding links to treatment and violence prevention has further contributed to this decline in throughput and increased length of stay, with no available evidence of improved public protection.

Previous studies which have used a predictive approach and relied entirely on retrospective, case register, and cross-sectional studies emphasise the importance of demographic, social, and previous criminal history factors, and report no association [24] or even negative associations between psychotic symptoms [25–27] and violent criminal behaviour. Similarly, that the association is largely or entirely explained by comorbid substance misuse or psychosocial adversity and environmental stressors more common in the lives of people with severe mental illness [28–30]. These factors undoubtedly increase risk of violence among persons with psychotic illness, together with lack of supervision after discharge, poor treatment compliance and treatment response. However, certain methodological shortcomings in earlier studies, particularly failure to investigate causal associations with dynamic factors, have led to over-emphasis of the role of pre-existing static risk factors. A key finding in this study was that, using the predictive approach, the HCR-20^v3^ failed to identify the strong, causal links we observed between appearance of symptoms of mental illness following discharge and subsequent violent behaviour. Reliance on findings from these previous studies could result in clinicians missing clear warning signs of future risk and failing to intervene, with serious consequences.

These findings have additional implications for future research. It has previously been observed that predictive accuracy is not influenced by large sample size, whether studies are retrospective, based on review of case files, or prospective, involving interviews by trained researchers [5, 6]. However, future research into causal associations will require adequate statistical power, using more complex methods within longitudinal studies, with multiple repeated measures, and using more appropriate statistical methods. A small number of previous studies have attempted to measure change over time using repeated ratings with actuarial instruments [31, 32] and SPJ [33–36]. However, predictive accuracy formed the basis of statistical analysis in these studies.

To manage risk and prevent violence, clinicians need to know which factors are causally related to that risk and ultimately which interventions are most effective. Alternatively, which factors are truly protective and mitigate that risk, and which might constitute the basis of a future intervention. No SPJ instrument currently specifies what interventions should be used on the basis of their ratings and training manuals leave such decisions to clinicians following a formulation [37]. The present study shows that in similar clinical samples, which largely include patients with psychotic illness, treatment interventions aimed to prevent deterioration in mental state, targeting symptoms of psychosis, associated affective symptoms and affect due to psychotic symptoms, together with associated behavioural disturbance, should be the main priority. Signs of increasing violent intent, behavioural instability, and stress/poor coping should result in urgent intervention.

Finally, our findings indicate that when training others in the use of such instruments, trainers will require extensive personal experience of the complex interplay between risk and protective factors, rather than just theoretical knowledge of managing risk, together with clinical skills in identifying risk factors, such as symptoms, which have causative associations with violence. Most importantly, they require experience of how to act effectively to prevent violence before it occurs.

## Supporting Information

S1 Appendix Statistical programming code. (DOCX)
