# Supplementary material for: Identifying Causal Risk Factors for Violence among Discharged Patients
Source: PLoS One. 2015 Nov 10;10(11):e0142493. doi: 10.1371/journal.pone.0142493 (PMC4640710; doi:10.1371/journal.pone.0142493)
Supplement: S1 Appendix — (DOCX) [file pone.0142493.s001.docx]

**S1 Supplementary appendix: statistical programming code**

*** TABLE 1. PREDICTIVE ACCURACY OF HCR-20^V3^ HISTORICAL ITEMS (MEASURED AT BASELINE)**

*** 2-level mixed effect model (repeatedness of measures/ subjects)**

foreach var of varlist hcr20h1_0 hcr20h2_0 hcr20h3_0 hcr20h4_0 hcr20h5_0 hcr20h6_0 hcr20h7_0 hcr20h8_0 hcr20h9_0 hcr20h10_0 Htotal {

display

desc `var'

display

somersd vio_ `var' cons1, trans(c) cluster(id)

display

lincom `var' - cons1

display

}

*** 3-level mixed effect model (repeatedness of measures/ subjects/ Trust)**

foreach var of varlist hcr20h1_0 hcr20h2_0 hcr20h3_0 hcr20h4_0 hcr20h5_0 hcr20h6_0 hcr20h7_0 hcr20h8_0 hcr20h9_0 hcr20h10_0 Htotal {

display

desc `var'

display

melogit vio_ c.`var' month1 || Trust: || id:, or

display

}

*** 3-level mixed effect model (repeatedness of measures/ subjects/ Trust) adjusted for age, gender, ethnicity, primary diagnosis at baseline**

foreach var of varlist hcr20h1_0 hcr20h2_0 hcr20h3_0 hcr20h4_0 hcr20h5_0 hcr20h6_0 hcr20h7_0 hcr20h8_0 hcr20h9_0 hcr20h10_0 Htotal {

display

desc `var'

display

melogit vio_ c.`var' month1 Age i.Gender i.Ethnicity diagrec month1 || Trust: || id:, or

display

}

*** TABLE 2. PREDICTIVE ACCURACY OF LAGGED VS. TEMPORAL PROXIMITY MODELS - AUC VALUES**

*** Lagged/predictive AUC**

*** HCR-20^v3^ - Clinical and risk management**

foreach var of varlist hcr20c1_la hcr20c2_la hcr20c3_la hcr20c4_la hcr20c5_la hcr20ccomp_la hcr20r1_la hcr20r2_la hcr20r3_la hcr20r4_la hcr20r5_la hcr20rcomp_la {

display

desc `var'

display

somersd vio_ `var' cons1, trans(c) cluster(id)

display

lincom `var' - cons1

display

}

*** SAPROF**

foreach var of varlist saprof1_la saprof2_la saprof3_la saprof4_la saprof5_la saprof6_la saprof7_la saprof8_la saprof9_la saprof10_la saprof11_la saprof12_la saprof13_la saprof14_la saprof15_la saprof16_la saprof17_la saprofcomp_la {

display

desc `var'

display

somersd vio_ `var' cons1, trans(c) cluster(id)

display

lincom `var' - cons1

display

}

*** Temporal proximity/causal AUC**

*** HCR-20^v3^ - Clinical and risk management**

foreach var of varlist hcr20c1_ hcr20c2_ hcr20c3_ hcr20c4_ hcr20c5_ hcr20ccomp_ hcr20r1_ hcr20r2_ hcr20r3_ hcr20r4_ hcr20r5_ hcr20rcomp_ {

display

desc `var'

display

somersd vio_ `var' cons1, trans(c) cluster(id)

display

lincom `var' - cons1

display

}

*** SAPROF**

foreach var of varlist saprof1_ saprof2_ saprof3_ saprof4_ saprof5_ saprof6_ saprof7_ saprof8_ saprof9_ saprof10_ saprof11_ saprof12_ saprof13_ saprof14_ saprof15_ saprof16_ saprof17_ saprofcomp_ {

display

desc `var'

display

somersd vio_ `var' cons1, trans(c) cluster(id)

display

lincom `var' - cons1

display

}

*** TABLE 3. STRENGTH OF ASSOCIATION IN LAGGED VS. TEMPORAL PROXIMITY MODELS – ODDS RATIOS**

*** Lagged/ predictive**

*** HCR-20^v3^ - Clinical**

forvalues i = 1(1)5 {

display

desc hcr20c`i'_la

display

melogit vio_ hcr20c`i'_la Age i.Gender i.Ethnicity diagrec month1 || Trust: || id:, or

display

}

melogit vio_ hcr20ccomp_la Age i.Gender i.Ethnicity diagrec month1 || Trust: || id:, or

*** HCR-20^v3^ - Risk management**

forvalues i = 1(1)5 {

display

desc hcr20r`i'_la

display

melogit vio_ hcr20r`i'_la Age i.Gender i.Ethnicity diagrec month1 || Trust: || id:, or

display

}

melogit vio_ hcr20rcomp_la Age i.Gender i.Ethnicity diagrec month1 || Trust: || id:, or

*** SAPROF**

forvalues i = 3(1)17 {

display

desc saprof`i'_la

display

melogit vio_ saprof`i'_la Age i.Gender i.Ethnicity diagrec month1 || Trust: || id:, or

display

}

*** Temporal proximity/ causal**

*** HCR-20^v3^ - Clinical**

forvalues i = 1(1)5 {

display

desc hcr20c`i'_

display

melogit vio_ hcr20c`i'_ Age i.Gender i.Ethnicity diagrec month1 || Trust: || id:, or

display

}

melogit vio_ hcr20ccomp_ Age i.Gender i.Ethnicity diagrec month1 || Trust: || id:, or

*** HCR-20^v3^ - Risk management**

forvalues i = 1(1)5 {

display

desc hcr20r`i'_

display

melogit vio_ hcr20r`i'_ Age i.Gender i.Ethnicity diagrec month1 || Trust: || id:, or

display

}

melogit vio_ hcr20rcomp_ Age i.Gender i.Ethnicity diagrec month1 || Trust: || id:, or

*** SAPROF**

forvalues i = 3(1)17 {

display

desc saprof`i'_

display

melogit vio_ saprof`i'_ Age i.Gender i.Ethnicity diagrec month1 || Trust: || id:, or

display

}

*** TABLE 4. INTER-ITEM CORRELATIONS**

*** HCR-20^v3^ historical**

spearman hcr20h1_0 hcr20h2_0 hcr20h3_0 hcr20h4_0 hcr20h5_0 hcr20h6_0 hcr20h7_0 hcr20h8_0 hcr20h9_0 hcr20h10_0, stats(rho obs p) bonferroni pw

*** HCR-20^v3^ clinical/ risk management**

*** Baseline**

spearman hcr20c1_0 hcr20c2_0 hcr20c3_0 hcr20c4_0 hcr20c5_0 hcr20r1_0 hcr20r2_0 hcr20r3_0 hcr20r4_0 hcr20r5_0, stats(rho obs p) bonferroni pw

*** 6 months follow-up**

spearman hcr20c1_6 hcr20c2_6 hcr20c3_6 hcr20c4_6 hcr20c5_6 hcr20r1_6 hcr20r2_6 hcr20r3_6 hcr20r4_6 hcr20r5_6, stats(rho obs p) bonferroni pw

*** 12 months follow-up**

spearman hcr20c1_12 hcr20c2_12 hcr20c3_12 hcr20c4_12 hcr20c5_12 hcr20r1_12 hcr20r2_12 hcr20r3_12 hcr20r4_12 hcr20r5_12, stats(rho obs p) bonferroni pw

*** SAPROF**

*** Baseline**

spearman saprof1_0 saprof2_0 saprof3_0 saprof4_0 saprof5_0 saprof6_0 saprof7_0 saprof8_0 saprof9_0 saprof10_0 saprof11_0 saprof12_0 saprof13_0 saprof14_0 saprof15_0 saprof16_0 saprof17_0, stats(rho obs p) bonferroni pw

*** 6 months follow-up**

spearman saprof3_6 saprof4_6 saprof5_6 saprof6_6 saprof7_6 saprof8_6 saprof9_6 saprof10_6 saprof11_6 saprof12_6 saprof13_6 saprof14_6 saprof15_6 saprof16_6 saprof17_6, stats(rho obs p) bonferroni pw

*** 12 months follow-up**

spearman saprof3_12 saprof4_12 saprof5_12 saprof6_12 saprof7_12 saprof8_12 saprof9_12 saprof10_12 saprof11_12 saprof12_12 saprof13_12 saprof14_12 saprof15_12 saprof16_12 saprof17_12, stats(rho obs p) bonferroni pw

*** TABLE 5. EXPLANATORY VARIABLES - HCR-20^V3^ CLINICAL AND RISK MANAGEMENT VARIABLES**

*** Model 0**

melogit vio_ Age i.Gender i.Ethnicity diagrec hcr20c1_ hcr20c2_ hcr20c3_ hcr20c4_ hcr20c5_ hcr20r1_ hcr20r2_ hcr20r3_ hcr20r4_ hcr20r5_ month1 || Trust: || id:, or

*** Model 1**

*** C: Lack of insight**

melogit vio_ Age i.Gender i.Ethnicity diagrec hcr20c1_ hcr20c2_ month1 || Trust: || id:, or

*** % explained**

di 100*(log(3.56) - log(1.59))/(log(3.56))

*** C: Symptoms of major mental disorder**

melogit vio_ Age i.Gender i.Ethnicity diagrec hcr20c3_ hcr20c2_ month1 || Trust: || id:, or

*** % explained**

di 100*(log(3.00) - log(1.68))/(log(3.00))

*** C: Treatment or supervision response**

melogit vio_ Age i.Gender i.Ethnicity diagrec hcr20c5_ hcr20c2_ month1 || Trust: || id:, or

*** % explained**

di 100*(log(2.92) - log(1.52))/(log(2.92))

*** R: Living situation**

melogit vio_ Age i.Gender i.Ethnicity diagrec hcr20r2_ hcr20c2_ month1 || Trust: || id:, or

*** % explained**

di 100*(log(1.81) - log(1.07))/(log(1.81))

*** R: Personal support**

melogit vio_ Age i.Gender i.Ethnicity diagrec hcr20r3_ hcr20c2_ month1 || Trust: || id:, or

*** % explained**

di 100*(log(2.16) - log(1.15))/(log(2.16))

*** R: Treatment or supervision response**

melogit vio_ Age i.Gender i.Ethnicity diagrec hcr20r4_ hcr20c2_ month1 || Trust: || id:, or

*** % explained**

di 100*(log(3.05) - log(1.44))/(log(3.05))

*** Model 2**

*** C: Lack of insight**

melogit vio_ Age i.Gender i.Ethnicity diagrec hcr20c1_ hcr20c4_ month1 || Trust: || id:, or

*** % explained**

di 100*(log(3.56) - log(1.55))/(log(3.56))

*** C: Symptoms of major mental disorder**

melogit vio_ Age i.Gender i.Ethnicity diagrec hcr20c3_ hcr20c4_ month1 || Trust: || id:, or

*** % explained**

di 100*(log(3.00) - log(1.38))/(log(3.00))

*** C: Treatment or supervision response**

melogit vio_ Age i.Gender i.Ethnicity diagrec hcr20c5_ hcr20c4_ month1 || Trust: || id:, or

*** % explained**

di 100*(log(2.92) - log(1.31))/(log(2.92))

*** R: Living situation**

melogit vio_ Age i.Gender i.Ethnicity diagrec hcr20r2_ hcr20c4_ month1 || Trust: || id:, or

*** % explained**

di 100*(log(1.81) - log(1.11))/(log(1.81))

*** R: Personal support**

melogit vio_ Age i.Gender i.Ethnicity diagrec hcr20r3_ hcr20c4_ month1 || Trust: || id:, or

*** % explained**

di 100*(log(2.16) - log(1.40))/(log(2.16))

*** R: Treatment or supervision response**

melogit vio_ Age i.Gender i.Ethnicity diagrec hcr20r4_ hcr20c4_ month1 || Trust: || id:, or

*** % explained**

di 100*(log(3.05) - log(1.22))/(log(3.05))

*** Model 3**

*** C: Lack of insight**

melogit vio_ Age i.Gender i.Ethnicity diagrec hcr20c1_ hcr20r5_ month1 || Trust: || id:, or

*** % explained**

di 100*(log(3.56) - log(2.13))/(log(3.56))

*** C: Symptoms of major mental disorder**

melogit vio_ Age i.Gender i.Ethnicity diagrec hcr20c3_ hcr20r5_ month1 || Trust: || id:, or

*** % explained**

di 100*(log(3.00) - log(1.88))/(log(3.00))

*** C: Treatment or supervision response**

melogit vio_ Age i.Gender i.Ethnicity diagrec hcr20c5_ hcr20r5_ month1 || Trust: || id:, or

*** % explained**

di 100*(log(2.92) - log(1.78))/(log(2.92))

*** R: Living situation**

melogit vio_ Age i.Gender i.Ethnicity diagrec hcr20r2_ hcr20r5_ month1 || Trust: || id:, or

*** % explained**

di 100*(log(1.81) - log(1.25))/(log(1.81))

*** R: Personal support**

melogit vio_ Age i.Gender i.Ethnicity diagrec hcr20r3_ hcr20r5_ month1 || Trust: || id:, or

*** % explained**

di 100*(log(2.16) - log(1.51))/(log(2.16))

*** R: Treatment or supervision response**

melogit vio_ Age i.Gender i.Ethnicity diagrec hcr20r4_ hcr20r5_ month1 || Trust: || id:, or

*** % explained**

di 100*(log(3.05) - log(1.50))/(log(3.05))

*** Model 4**

*** C: Lack of insight**

melogit vio_ Age i.Gender i.Ethnicity diagrec hcr20c1_ hcr20c2_ hcr20c4_ hcr20r5_ month1 || Trust: || id:, or

*** % explained**

di 100*(log(3.56) - log(0.99))/(log(3.56))

*** C: Symptoms of major mental disorder**

melogit vio_ Age i.Gender i.Ethnicity diagrec hcr20c3_ hcr20c2_ hcr20c4_ hcr20r5_ month1 || Trust: || id:, or

*** % explained**

di 100*(log(3.00) - log(1.07))/(log(3.00))

*** C: Treatment or supervision response**

melogit vio_ Age i.Gender i.Ethnicity diagrec hcr20c5_ hcr20c2_ hcr20c4_ hcr20r5_ month1 || Trust: || id:, or

*** % explained**

di 100*(log(2.92) - log(0.89))/(log(2.92))

*** R: Living situation**

melogit vio_ Age i.Gender i.Ethnicity diagrec hcr20r2_ hcr20c2_ hcr20c4_ hcr20r5_ month1 || Trust: || id:, or

*** % explained**

di 100*(log(1.81) - log(0.89))/(log(1.81))

*** R: Personal support**

melogit vio_ Age i.Gender i.Ethnicity diagrec hcr20r3_ hcr20c2_ hcr20c4_ hcr20r5_ month1 || Trust: || id:, or

*** % explained**

di 100*(log(2.16) - log(0.96))/(log(2.16))

*** R: Treatment or supervision response**

melogit vio_ Age i.Gender i.Ethnicity diagrec hcr20r4_ hcr20c2_ hcr20c4_ hcr20r5_ month1 || Trust: || id:, or

*** % explained**

di 100*(log(3.05) - log(0.71))/(log(3.05))

*** TABLE 6. EXPLANATORY VARIABLES - SAPROF**

*** Model 0**

melogit vio_ Age i.Gender i.Ethnicity diagrec saprof3_ saprof4_ saprof5_ saprof6_ saprof7_ saprof8_ saprof9_ saprof10_ saprof11_ saprof12_ saprof13_ saprof14_ saprof15_ saprof16_ saprof17_ month1 || Trust: || id:, or

*** Model 1**

*** Empathy**

melogit vio_ Age i.Gender i.Ethnicity diagrec saprof3_ saprof5_ month1 || Trust: || id:, or

*** % explained**

di 100*(log(0.32) - log(0.62))/(log(0.32))

*** Coping**

melogit vio_ Age i.Gender i.Ethnicity diagrec saprof4_ saprof5_ month1 || Trust: || id:, or

*** % explained**

di 100*(log(0.21) - log(0.65))/(log(0.21))

*** Work**

melogit vio_ Age i.Gender i.Ethnicity diagrec saprof6_ saprof5_ month1 || Trust: || id:, or

*** % explained**

di 100*(log(0.52) - log(0.80))/(log(0.52))

*** Leisure activities**

melogit vio_ Age i.Gender i.Ethnicity diagrec saprof7_ saprof5_ month1 || Trust: || id:, or

*** % explained**

di 100*(log(0.36) - log(0.66))/(log(0.36))

*** Financial management**

melogit vio_ Age i.Gender i.Ethnicity diagrec saprof8_ saprof5_ month1 || Trust: || id:, or

*** % explained**

di 100*(log(0.50) - log(1.08))/(log(0.50))

*** Motivation for treatment**

melogit vio_ Age i.Gender i.Ethnicity diagrec saprof9_ saprof5_ month1 || Trust: || id:, or

*** % explained**

di 100*(log(0.34) - log(0.85))/(log(0.34))

*** Attitudes towards authority**

melogit vio_ Age i.Gender i.Ethnicity diagrec saprof10_ saprof5_ month1 || Trust: || id:, or

*** % explained**

di 100*(log(0.27) - log(0.60))/(log(0.27))

*** Life goals**

melogit vio_ Age i.Gender i.Ethnicity diagrec saprof11_ saprof5_ month1 || Trust: || id:, or

*** % explained**

di 100*(log(0.42) - log(0.87))/(log(0.42))

*** Medication**

melogit vio_ Age i.Gender i.Ethnicity diagrec saprof12_ saprof5_ month1 || Trust: || id:, or

*** % explained**

di 100*(log(0.41) - log(0.90))/(log(0.41))

*** Social network**

melogit vio_ Age i.Gender i.Ethnicity diagrec saprof13_ saprof5_ month1 || Trust: || id:, or

*** % explained**

di 100*(log(0.41) - log(0.70))/(log(0.41))
